# Ultrasonic Array-Based Multi-Source Fusion Indoor Positioning Technology

**DOI:** 10.3390/s24206641

**Published:** 2024-10-15

**Authors:** Cong Li, Chenning Zhang, Bing Chen, Shaojian Xu, Luping Xu, Bo Yan

**Affiliations:** 1 National Key Laboratory of Science and Technology on Space Microwave, Xi’an 710100, China; lic1@cast504.com; 2 School of Aerospace Science and Technology, Xidian University, Xi’an 710126, China; 22131214171@stu.xidian.edu.cn (C.Z.); lpxu@mail.xidian.edu.cn (L.X.); 3 Laboratory of Transport Safety and Emergency Technology, Transport Planning and Research Institute, Beijing 100029, China; chenbing@tpri.org.cn; 4 Beijing Leiyin Electronic Technology Development Co., Ltd., Beijing 100070, China; xushaojian@139.com

**Keywords:** indoor positioning, ultrasonic, TDoA, positioning accuracy, GDOP

## Abstract

Underground mining involves numerous risks, such as collapses, gas leaks, and explosions, posing significant threats to worker safety. In this work, we develop an indoor localization system that uses Bluetooth for coarse positioning and ultrasonic arrays for precision calibration. This system is particularly useful for automated mining operations in underground environments where satellite positioning signals are unavailable. The indoor localization system consists of ultrasonic receiver arrays and an improved multi-transmitter-multi-receiver algorithm, enabling accurate localization within the mining environment. Geometric Dilution of Precision (GDOP) analysis is incorporated to optimize the network layout, and an inertial navigation module is integrated to track the posture of moving objects, enabling precise trajectory determination over large areas, such as coal mines. In the experiment, three traditional methods were compared, and the proposed tracking approach demonstrated a positioning accuracy within 10 cm, reducing error by 20% compared to conventional techniques. This high-precision indoor localization method proves beneficial for underground mining applications.

## 1. Introduction

With the rapid advancement of mechanical automation, the adoption of intelligent and automated processes has become widespread across large-scale mining operations. Enhancing operational efficiency and safeguarding worker safety necessitates the development and implementation of a highly efficient localization system designed specifically to support mining activities and protect personnel. Such a system must deliver high localization precision within complex environments and be capable of accurately tracking the orientation and position of mobile equipment. However, current methods for detecting orientation and position lack the necessary precision and are highly susceptible to environmental factors, leading to limited application success in specialized environments like mines [1].

**Figure 1:**
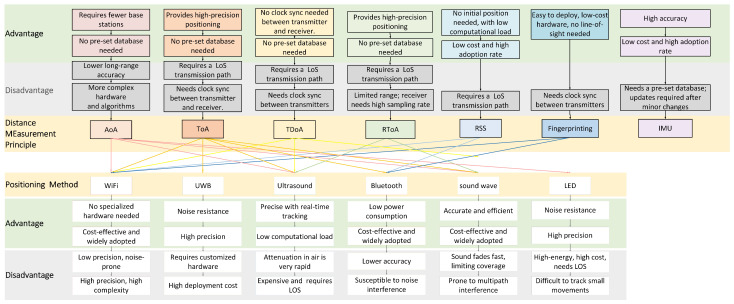
Distance Measurement Principle and Positioning Principle (RToA stands for “Round-Trip Time of Arrival”).

In contemporary society, indoor positioning technology has significantly enhanced the functionality and interactivity of various spaces such as indoor navigation [2,3]. The essence of this technology lies in its ability to provide precise location data within indoor environments, where traditional Global Positioning Systems (GPS) falter due to signal attenuation and obstruction [2]. The devices for the indoor positioning include Wireless Fidelity (Wi-Fi), Radio Frequency Identification (RFID), ultrasonic positioning systems, Inertial Measurement Unit (IMU), Ultra-Wideband (UWB) technology, and Bluetooth [4]. Figure 1 organizes the current state of research in indoor positioning in recent years.

This paper introduces an innovative indoor positioning system that integrates ultrasonic arrays, Bluetooth, and inertial navigation modules. The proposed system addresses the limitations of existing technologies such as AoA (Angle of Arrival), ToA [5] (Time of Arrival), RSS [6] (Received Signal Strength), and IMU [7,8] (Inertial Measurement Unit). By combining these technologies, our method achieves high-precision positioning with reduced costs and enhanced robustness, particularly in complex environments like underground mines where traditional systems often fail. The experimental results demonstrate that our approach improves positioning accuracy by 20%, achieving an error margin within 10 cm, making it an ideal solution for applications requiring reliable and precise indoor localization.

As illustrated in Figure 1, high-precision positioning techniques such as AoA [9,10] and ToA [11] typically demand stringent hardware capabilities, including precise clock synchronization between devices and the ability to handle high computational loads. These requirements significantly elevate implementation costs, thereby limiting the widespread adoption of these technologies in everyday applications. Conversely, while less accurate, more cost-effective solutions like RSS and IMU [12,13] are commonly used in indoor positioning due to their affordability.

Bluetooth, ultrasonic, and inertial navigation technologies each offer distinct advantages and drawbacks when compared to other indoor positioning methods, and their integration can create more robust systems.

Bluetooth is widely favored due to its ubiquity, low energy consumption, and cost-effectiveness. It is easy to deploy in various environments, making it suitable for applications like navigation and asset tracking [14]. However, Bluetooth signals are susceptible to interference from physical barriers and multipath effects, reducing accuracy, particularly in complex environments [15]. Although it provides convenient indoor positioning, Bluetooth alone struggles with high-precision demands, especially in GPS-level accuracy scenarios.

Ultrasonic positioning systems, on the other hand, provide higher accuracy and are immune to electromagnetic interference, making them reliable in certain environments. However, their effectiveness is heavily dependent on line-of-sight conditions and can be compromised by obstructions, reflections, and variations in sound speed, limiting their range and precision.

Inertial Measurement Units (IMU) offer key advantages, such as independence from external signals, allowing them to operate autonomously in GPS-denied environments, including underground or indoor spaces. IMU provide continuous real-time tracking of position and orientation, ensuring uninterrupted data flow. However, their main limitation is error accumulation over time, leading to drift. Furthermore, IMU can only track relative movements and cannot provide absolute positioning, necessitating integration with other technologies to enhance accuracy and reliability.

Building on an analysis of the strengths and weaknesses of these technologies and the comparative results in Figure 1 [16,17], this article proposes a fusion design that integrates Bluetooth [18], ultrasonic, and inertial navigation modules. The goal is to achieve low-cost, high-precision, long-range, and durable positioning in mining environments. By balancing cost and effectiveness, the proposed system aims to create an efficient and economical framework for monitoring and ensuring safety in mining operations, leveraging the synergistic integration of Bluetooth, ultrasonic, and IMU technologies.

Although Bluetooth alone has limitations in achieving high precision, especially in environments with significant signal reflections and obstructions, recent research has shown that integrating it with technologies such as IMU, Pedestrian Dead Reckoning (PDR) [19], UWB [20], and ultrasonic positioning can effectively address these shortcomings. IMU and PDR provide continuous tracking of movement, helping to reduce drift and improve positioning accuracy over time, while UWB and ultrasonic systems offer higher precision and robustness against interference. This combination creates a more reliable and accurate positioning system, particularly in complex environments where Bluetooth signals face challenges.

**Figure 2:**
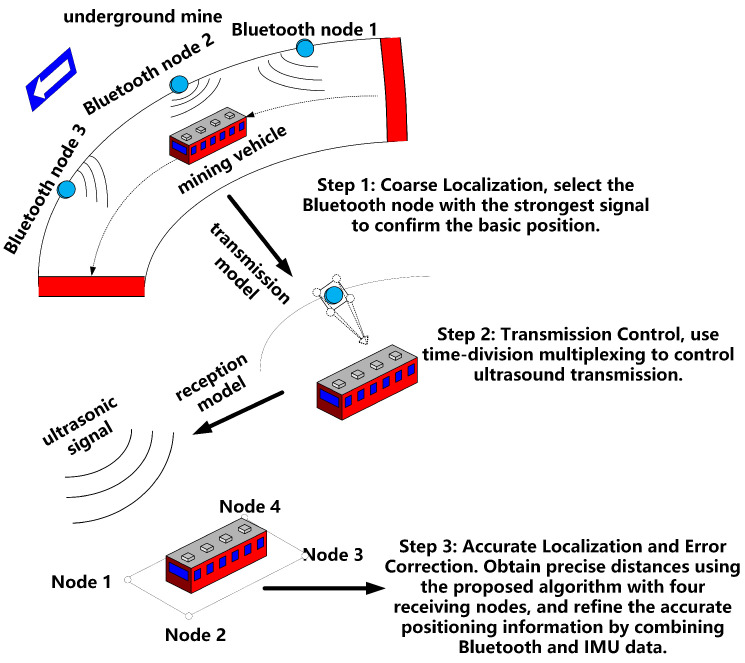
Overall system concept.

As depicted in Figure 2, Bluetooth beacons are strategically placed every 10 m along the track to provide rough positioning and to signal the system when to transition into the ultrasonic range. A custom-developed ultrasonic algorithm is then employed for precise correction. Given the inherent limitations of Bluetooth positioning and its tendency to accumulate errors, an ultrasonic system is deployed to mitigate these issues. This system corrects cumulative motion errors, accurately determines the target’s orientation, and ensures stable and precise positioning. Simultaneously, an inertial navigation module is utilized to optimize the trajectory, enabling position tracking and trajectory mapping within the mine. This integration supports machinery operation monitoring and enhances personnel safety.

This paper delves into ultrasonic localization technology by designing a multi-transmitter-multi-receiver module. The integration of this module with a multi-receiver collection system is aimed at achieving high-precision spatial positioning.

**Figure 3:**
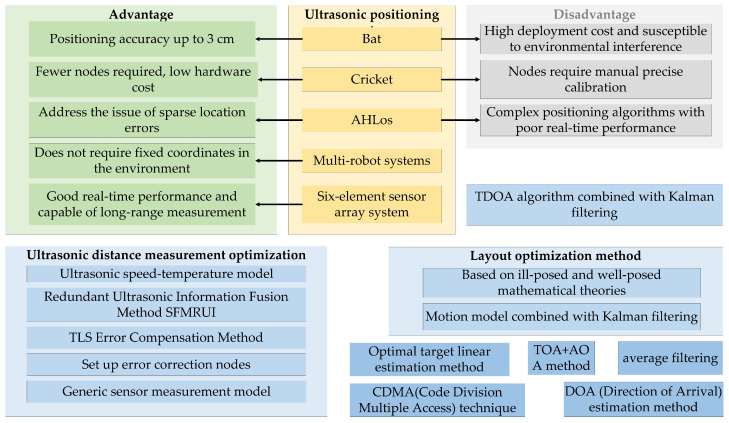
Current state of research.

The development history of ultrasound is shown in Figure 3. Since the development of the Bat system by AT&T Labs in 1999, ultrasonic technology has been widely applied in indoor positioning. The Bat system utilizes ultrasonic transmitters, receivers, and a master server to locate moving targets by periodically emitting signals, achieving an accuracy of up to 3 cm. However, ultrasonic signals are susceptible to environmental interference, leading to decreased system stability, and the extensive use of receivers significantly increases deployment costs. In 2000, the Cricket system developed by the Massachusetts Institute of Technology combined radio frequency communication with ultrasonic technology, measuring distances using Time Difference of Arrival (TDoA) and determining coordinates through trilateration. Although this system reduced the number of sensors, it has stringent requirements for the positioning of receiving nodes, and positioning errors can easily arise with changes in the environment or network coverage. In 2003, the AHLos system developed by the University of California, Los Angeles, improved upon the Cricket system by implementing a distributed architecture and maximum likelihood estimation for positioning. However, the complexity of the algorithms makes real-time positioning challenging. In 2014, various algorithms, such as Total Least Squares (TLS) and minimax algorithms, were applied in ultrasonic positioning to compensate for ranging errors and calculate target locations. Additionally, the integration of Kalman filtering with related motion models in 2015 further enhanced the system’s real-time performance and accuracy.

In conjunction with the results illustrated in Figure 3, the limitations and shortcomings of indoor ultrasonic research methods reveal several areas in need of improvement. First, there is the issue of equipment cost. The integrated nature of ultrasonic modules can significantly elevate costs, particularly when achieving frequency separation and coding, which are essential in practical applications. To address this, a time-division mode is employed, utilizing time differences to determine signal arrival.

Secondly, in real-world applications, many algorithms used for address screening [21] and coding [22] assignments result in substantial data processing, which can impact real-time performance. Additionally, the system exhibits notable time redundancy, and the excessive computational demands can lead to increased energy consumption [23], thereby limiting the long-term stability and feasibility of these algorithms in practical environments [24].

Regarding layout processing, most current algorithms rely on existing strategies, such as circular, spiral, and polygonal layouts. However, these traditional methods can struggle to adapt to and meet the needs of complex indoor environments.

To address these challenges, this study proposes the design of a multi-transmitter-multi-receiver ultrasonic calculation system and the construction of a network to achieve high-precision positioning within a defined range. The goal is to enable precise positioning and attitude measurement in complex environments. The core concept centers on an ultrasonic system, with the primary focus on constructing a receiving array composed of four receivers, used to calculate the exact distance to the transmitters. Specifically, using a Bluetooth beacon as a reference point, four transmitters A,B,C,D are placed around the beacon, forming a square spatial layout.

**Initial Bluetooth Beacon Positioning:** First, Bluetooth beacon technology is employed to estimate the target’s approximate location range.

**Ultrasonic Distance Calculation:** The input distance measurement data set *L* is represented as:

(1) $$L=\left\{\left(a_{i},b_{i},c_{i},d_{i}\right)|i=1,2,3,4\right\}$$

Here, $a_{i},b_{i},c_{i},d_{i}$ represent the distances measured by four receivers from four respective transmitters.

The input data *A* is processed using a custom algorithm to compute the distance vector *X*, representing the distance from the transmitter to the center of the receiver array:

(2) $$X=A\cdot L=\left(x_{i}|i=1,2,3,4\right)$$

**Coordinate and Attitude Calculation:** Subsequently, the distance vector *X* is processed using a trilateration algorithm. By applying matrix *B*, the specific spatial coordinates of the target (x,y,z) and the attitude angles (ψ,θ,ϕ) are calculated:

(3) $$B\cdot X=\left(x,y,z,\psi ,\theta ,\phi \right)$$

Here, ψ is the yaw angle, θ is the pitch angle, and ϕ is the roll angle.

**Inertial Navigation Trajectory Correction:** In the final step, the inertial navigation module is utilized to refine the preliminary trajectory, thereby ensuring the continuous and accurate representation of both positioning and attitude data.

In contrast to conventional techniques, the method introduced in this research mitigates system errors by filtering the confidence levels of measurement values, thereby enhancing the overall stability of the system. While most traditional technologies are typically limited to performing either positioning or orientation tasks independently, the matrix-solving approach adopted in this study enables the simultaneous execution of both tasks within a three-dimensional space. This dual capability offers significant advantages for guiding and planning the paths of mining machinery and personnel movements. Moreover, by optimizing the GDOP layout to identify the optimal configuration, this approach significantly enhances positioning accuracy and supports continuous large-scale positioning within the mine environment. Additionally, the experimental setup employs low-cost equipment modules, which not only facilitate extensive deployment but also ensure low power consumption, making them ideal for long-term operation and monitoring.

The paper is structured as follows: Section 2 provides an overview of the system, including the system model and measurement model. Section 3 outlines the specific steps of the proposed methodology. Section 4 showcases the method’s advantages through experiments conducted in complex indoor settings. Finally, Section 5 discusses the limitations of the approach and suggests potential directions for future research.

## 2. System Model

This system achieves coarse positioning through Bluetooth, while the ultrasonic array employs the TDoA algorithm for precise calibration. The GDOP optimization algorithm is used to adjust the transmitter layout, minimizing positioning errors. Compared to traditional ToA or RSS methods, the system’s multi-transmitter-multi-receiver model integrates TDoA positioning and an inertial navigation module, significantly enhancing positioning accuracy in complex environments.

Time Difference of Arrival (TDoA) is a positioning technique that estimates the distance between a receiver and multiple senders by analyzing the time differences in signal arrival from various sources. Unlike Time of Arrival (ToA) or Time of Flight (ToF) methods, TDoA does not necessitate knowledge of the exact time at which the signals were transmitted by the sender. In the TDoA approach, the time difference between when the signal reaches the receiver from the i-th sender and the j-th sender is represented as $T_{D(i,j)}$. The corresponding difference in physical distance can then be determined using the following formula:

(4) $$L_{D(i,j)}=c\cdot T_{D(i,j)}$$

Here, *c* represents the speed of the signal in the medium.

Given that the signal’s speed within the medium is both known and constant, there is a direct correlation between the flight distance and the flight time. When the transmitter times are either synchronized or the intervals are known, the flight time can be translated into the signal’s arrival time. Consequently, measuring the time differences between the signals emitted by various transmitters (Tx) as they reach the receiver (Rx) is sufficient to determine the associated distance differences:

(5) $$L_{D(i,j)}=\sqrt{{\left(x-x_{i}\right)}^{2}+{\left(y-y_{i}\right)}^{2}+{\left(z-z_{i}\right)}^{2}}-\sqrt{{\left(x-x_{j}\right)}^{2}+{\left(y-y_{j}\right)}^{2}+{\left(z-z_{j}\right)}^{2}}$$

In this context, ($x_{i},y_{i},z_{i}$) are the coordinates corresponding to the i-th sender, while (x,y,z) are the coordinates of the receiver. The term $L_{D(i,j)}$ defines a hyperbolic equation. To accurately pinpoint the receiver’s position, it is essential to calculate the time differences of arrival from at least three senders. The receiver’s position is then estimated by finding the intersection of the hyperbolas formed by the TDoA measurements.

### 2.1. Problem Analysis

**Figure 4:**
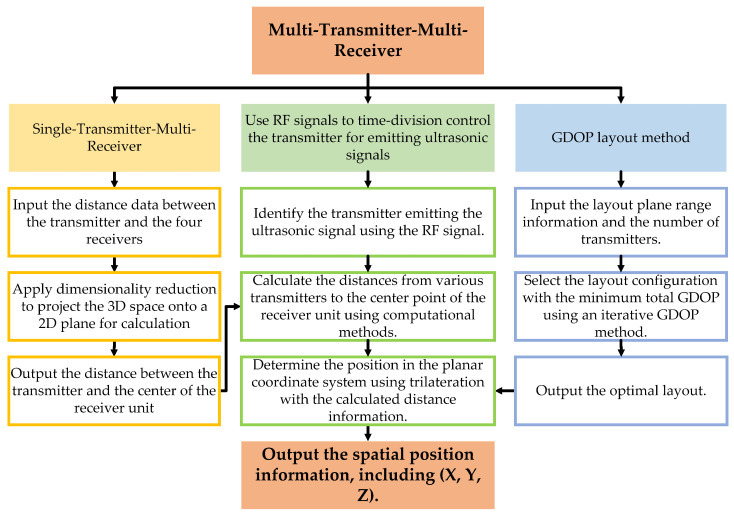
Ultrasonic positioning system calculation process (RF stands for “Radio Frequency”).

This study focuses on the design and development of a high-precision indoor positioning system, which integrates multiple transmitters and receiver units to form a multi-transmitter-multi-receiver network architecture, akin to satellite positioning systems. The objective is to attain precise positioning with an error margin of no more than 5 cm within a confined indoor setting. The overall process flow is depicted in Figure 4.

#### 2.1.1. The Single-Transmitter-Multi-Receiver Model

Building on the principles of TDoA ultrasonic ranging, we have developed a physical model for a single-transmitter-multi-receiver setup and introduced a novel approach. This method begins by reducing dimensionality through solving positional information on a two-dimensional plane, which is subsequently extended to a three-dimensional space. This approach streamlines the positioning process and improves accuracy.

#### 2.1.2. The Multi-Transmitter-Multi-Receiver System

In this step, we will apply the principles of the single-receiver-multi-transmitter model in combination with the trilateration method. Through the application of time-division multiplexing, this model is integrated into a multi-transmitter-multi-receiver system. The trilateration technique is employed to calculate absolute position information based on the relative data provided by each beacon.

#### 2.1.3. Transmitter Network Layout Optimization

This section introduces Geometric Dilution of Precision (GDOP) as a fundamental concept in optimizing transmitter network layouts. The approach aims to minimize the total GDOP value, which serves as the objective function in an iterative optimization algorithm, to determine the most effective beacon arrangement.

### 2.2. Algorithm Framework

#### 2.2.1. Construction of a Single-Transmitter-Multi-Receiver Model Based on TDoA

**Figure 5:**
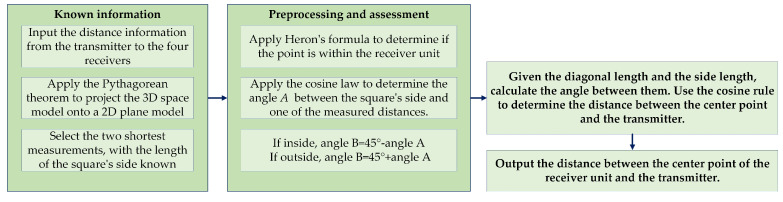
Single-transmitter-multi-receiver calculation flowchart.

The TDoA method eliminates the need for temporal synchronization between the transmitter and receiver, focusing instead on synchronization among multiple transmitters. This method is robust against single-signal interference since it relies on TDoA at multiple receivers, allowing compensation for disturbances in some paths. TDoA outperforms ToA in Non-Line-of-Sight (NLOS) environments by leveraging time difference information from multiple receivers to mitigate NLOS-induced errors. Consequently, this paper presents a one-to-many ultrasonic array model based on these principles, with the solution process illustrated in Figure 5.

#### 2.2.2. The Multi-Transmitter-Multi-Receiver Based Trilateration System

**Figure 6:**
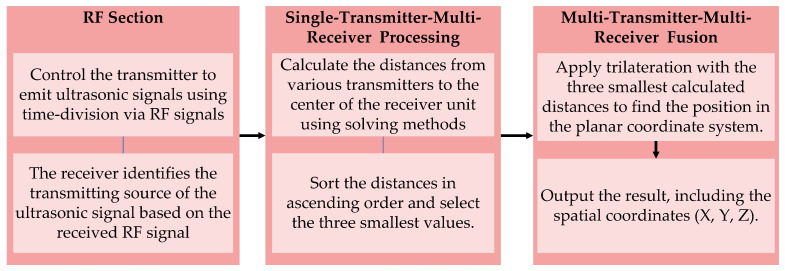
Multi-transmitter-multi-receiver fusion.

In the model involving multiple transmitters and a single receiver, common methods include trilateral positioning, triangulation, and maximum likelihood estimation. The trilateral positioning algorithm is favored for its simplicity and computational ease, providing accurate position coordinates when ultrasonic ranging is precise. The maximum likelihood estimation method, while effective in minimizing systematic errors, can suffer from increased algorithmic complexity, potentially leading to slower system response times. The triangulation method, which relies on angle measurements, can be difficult to implement in practical scenarios. For systems with multiple ultrasonic beacons, the approach prioritizes selecting the three nearest beacons and applying the trilateral positioning method to achieve effective indoor localization. The calculation flow for this integrated system model is depicted in Figure 6.

#### 2.2.3. Transmitter Network Layout Optimization Based on GDOP

**Figure 7:**
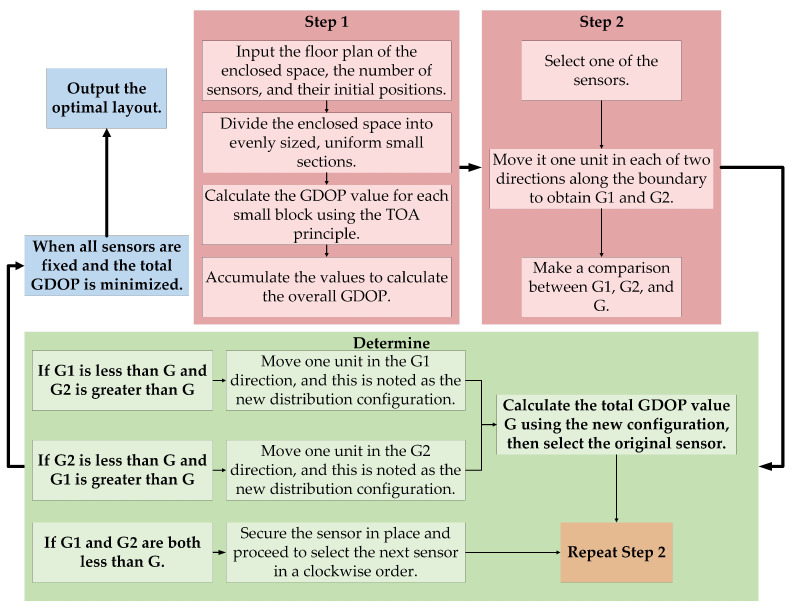
GDOP layout flowchart.

The solution process is illustrated in Figure 7. The GDOP factor is a crucial metric for evaluating the spatial relationships between sensor transmitters and receivers in an indoor positioning system. GDOP measures how the positional relationship between the target and the sensors affects overall positioning accuracy. By calculating the GDOP factor, one can gauge the reliability and precision of the sensor network, guiding the optimization of sensor deployment locations and quantities. This optimization enhances the accuracy and reliability of indoor positioning systems.

### 2.3. Summary of This Chapter

To validate the uniqueness of this system, we compared it with several common indoor positioning technologies. Although traditional ToA methods can provide high accuracy, they rely on precise clock synchronization, which significantly increases hardware costs. While RSS technology is more cost-effective, it is highly susceptible to multipath effects and signal attenuation, limiting its accuracy. By introducing TDoA and GDOP optimization strategies, this system effectively improves positioning accuracy in complex environments, while the use of low-cost ultrasonic modules helps control overall system costs.

Compared to existing multi-source fusion methods, this research innovatively combines Bluetooth coarse positioning, ultrasonic precision calibration, and an inertial navigation module. This not only improves positioning accuracy but also enables stable positioning performance under signal obstruction through multipath analysis and reflected signal estimation.

In experimental comparisons, the system’s positioning error was kept within 10 cm in environments without obstructions, and under partial obstruction, positioning error was limited to ±50 mm through reflected signal analysis and multi-source data fusion. In contrast, traditional single-transmitter-single-receiver models had positioning errors of up to 100 mm under obstruction, highlighting the significant advantage of this system in complex environments.

## 3. Data Processing

The design is divided into three key parts:

**One-to-Many Transmitter Model:** This involves setting up a model where a single transmitter communicates with multiple receivers.

**Fusion of Multiple Transmitter–Receiver Models:** This step integrates various transmitter–receiver models to improve system accuracy.

**Transmitter Layout Optimization:** Focuses on optimizing the layout of transmitters to enhance overall positioning performance.

The entire system combines these models with an optimization algorithm for the layout, achieving absolute positioning through the multi-transmitter-receiver model. The layout optimization improves ranging accuracy and positioning performance.

Ultrasonic transmitters are strategically installed at fixed positions within the building to cover the entire positioning area. The number and locations of these nodes are based on the building’s structure and positioning needs. Given the range limitations of ultrasonic positioning, a multi-transmitter-receiver model is established, with transmitters placed at the four upper corners of the area to ensure that targets within the region can receive the ultrasonic signals effectively.

### 3.1. Single-Transmitter-Multi-Receiver Model

#### 3.1.1. Ultrasonic Distance Measurement Processing

**Figure 8:**
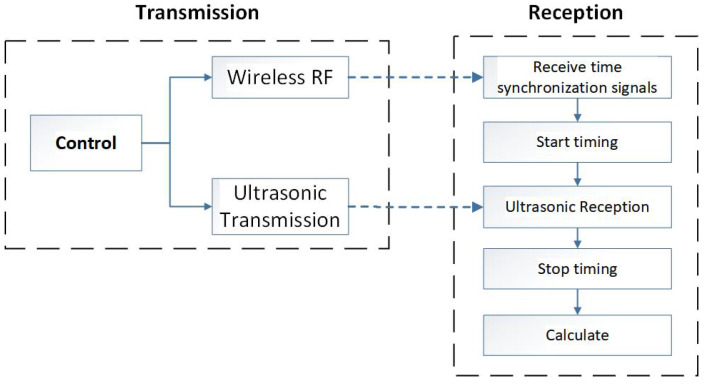
Through-Beam Ultrasonic Ranging Method.

The principle of ultrasonic distance measurement is shown in Figure 8. While reflective ranging is simple and convenient, it has a notable limitation: it cannot reliably verify if the detected target is the intended one. To address this limitation, we utilize radiation-based ranging. This approach generally employs specialized ultrasonic transducers. The process for radiation-based ranging is as follows: The main control unit initiates the emission of ultrasonic waves and concurrently transmits a time-synchronized radio frequency (RF) signal. Once the receiver detects the synchronized RF signal, it starts timing and halts when the ultrasonic signal is received. The distance between the transmitter and receiver is calculated by multiplying the speed of sound by the time interval measured. This technique provides precise distance measurements between devices, thereby effectively identifying the target object.We define the measured distance as

(6) L=V·Δt

The time difference between the ultrasonic propagation and the radio frequency (RF) transmitter, denoted as Δt, represents the interval between the arrival of the ultrasonic signal and the synchronized RF signal:

(7) $$\Delta t=t_{ultrasonic}-t_{rf}$$

The radio frequency signal travels from the transmitter to the receiver at the speed of light, while the ultrasonic wave propagates at approximately 340 m/s. Given that the speed of light is roughly $10^{6}$ times greater than the speed of sound, the time $t_{rf}$ required for the radio frequency signal is negligible in comparison. Therefore, the distance *L* can be calculated as

(8) $$L=V\cdot t_{ultrasonic}$$

Here, $t_{ultrasonic}$ represents the propagation time of the ultrasonic wave from emission to receiver, as recorded by the timer at the receiver end.

#### 3.1.2. Relative Distance Positioning with Single-Transmitter-Multi-Receiver

**Figure 9:**
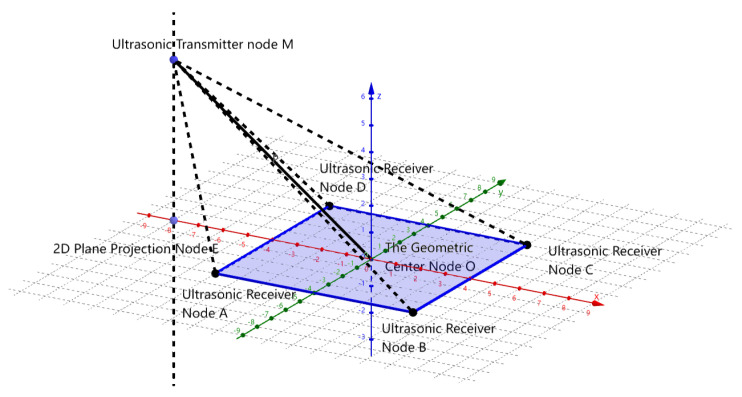
Dimensionality reduction mathematical model for the single-transmitter-multi-receiver system.

This method determines the relative position of the transmitter with respect to the receiver array. The range model is illustrated in Figure 9: Point *M* represents the transmitter, and ABCD denotes the receiver array. By projecting point *M* onto a two-dimensional plane, the relative distance and angle between the projection point *E*, the center of the array *O*, and *M* can be calculated using an advanced algorithm.

### 3.2. Multi-Transmitter-Multi-Receiver Model

#### 3.2.1. Absolute Positioning Processing with Multi-Transmitter-Single-Receiver

In this positioning experiment, the trilateral positioning method is employed. Assume there are transmitters 1, 2, and 3 positioned around receiver *X*. These represent the three ultrasonic emitters involved in the positioning process, while *X* denotes the multi-receptor ultrasonic receiving array. By applying TDoA principle, the distances from the three base stations to the target *X* are measured as $r_{1}$, $r_{2}$, $r_{3}$. These distance measurements are then used to solve for the coordinates of the target *X*. Geometrically, this involves drawing three circles based on the distance information and the centers of the base stations. The intersection of these circles determines the position of the target.

Let $R_{i}\left(i=1,2,3\right)$ represent the distances from the moving node to the projection points of the ultrasonic beacons on the ground. Given the distances $R_{1}$, $R_{2}$, $R_{3}$, the position coordinates of the moving node (x,y) can be determined by finding the intersection point of the circles centered at the projection points of the beacons.

(9) $$\left\{\begin{array}{c} {\left(x_{1}-x\right)}^{2}-{\left(y_{1}-y\right)}^{2}=R_{1}^{2}\\ {\left(x_{2}-x\right)}^{2}-{\left(y_{2}-y\right)}^{2}=R_{2}^{2}\\ {\left(x_{3}-x\right)}^{2}-{\left(y_{3}-y\right)}^{2}=R_{3}^{2} \end{array}\right.$$

The range value between the indoor ceiling ultrasonic beacon and the ground moving node is $L_{i}=\left(i=1,2,3\right)$. Let the altitude of the aerial ultrasonic beacon from the moving node be *H*, then by $R_{i}^{2}=L_{i}^{2}-H_{i}^{2}$. Let $L_{i}=\left(i=1,2,3\right)$ represent the distance between the indoor ceiling ultrasonic beacons and the moving node on the ground. Define *H* as the altitude of the ultrasonic beacon above the moving node. The relationship between $L_{i}$, *H*, and $R_{i}$ (where $R_{i}$ is the horizontal distance from the moving node to the projection of the beacon on the ground) can be expressed as $R_{i}^{2}=L_{i}^{2}-H_{i}^{2}$

(10) $$\left\{\begin{array}{c} x=\frac{\left(L_{1}^{2}-L_{2}^{2}+y_{2}^{2}-y_{1}^{2}+x_{2}^{2}-x_{1}^{2}\right)\left(y_{3}-y_{1}\right)}{2[\left(x_{2}-x_{1}\right)\left(y_{3}-y_{1}\right)-\left(x_{3}-x_{1}\right)\left(y_{2}-y_{1}\right)]}-\frac{\left(L_{1}^{2}-L_{3}^{2}+y_{3}^{2}-y_{1}^{2}+x_{3}^{2}-x_{1}^{2}\right)\left(y_{2}-y_{1}\right)}{2[\left(x_{2}-x_{1}\right)\left(y_{3}-y_{1}\right)-\left(x_{3}-x_{1}\right)\left(y_{2}-y_{1}\right)]}\\ y=\frac{\left(L_{1}^{2}-L_{2}^{2}+y_{2}^{2}-y_{1}^{2}+x_{2}^{2}-x_{1}^{2}\right)\left(x_{3}-x_{1}\right)}{2[\left(x_{3}-x_{1}\right)\left(y_{2}-y_{1}\right)-\left(x_{2}-x_{1}\right)\left(y_{3}-y_{1}\right)]}-\frac{\left(L_{1}^{2}-L_{3}^{2}+y_{3}^{2}-y_{1}^{2}+x_{3}^{2}-x_{1}^{2}\right)\left(x_{2}-x_{1}\right)}{2[\left(x_{3}-x_{1}\right)\left(y_{2}-y_{1}\right)-\left(x_{2}-x_{1}\right)\left(y_{3}-y_{1}\right)]} \end{array}\right.$$

#### 3.2.2. Multi-Transmitter-Multi-Receiver Systems

By combining positioning data from multi-transmitter-multi-receiver technologies, the system can more effectively mitigate errors introduced by multipath effects in complex environments. This integration enhances the accuracy and reliability of the device’s location determination.

### 3.3. System Layout Models

The ultrasonic positioning system described in this paper utilizes a trilateration algorithm with three ultrasonic sensors for positioning. However, three sensors may be insufficient to cover the entire localization area. In such cases, optimizing the layout of only these three sensors is inadequate; instead, a comprehensive optimization of the sensor layout is required. To achieve this, the system employs the minimum sum of GDOP across all reference points in the area as the evaluation criterion. The x- and y-coordinates of the ultrasonic sensors are iteratively adjusted to find the optimal layout. The specific implementation process is as follows:

#### 3.3.1. Model Building

Define the initial positions of the ultrasonic sensors and reference points. Establish a rectangular coordinate system within a region of dimensions N×M, where the *x*-axis extends along the boundary of length *N* and the *y*-axis extends along the boundary of length *M*. Place *S* ultrasonic sensors at the boundary of the area with initial 2D coordinates ($\left(x_{1},y_{1}\right),\left(x_{2},y_{2}\right),\cdots ,\left(x_{s},y_{s}\right)$). The enclosed space is divided into cells of side length *d*, with each cell represented by coordinates (i,j), where *i* and *j* denote the horizontal and vertical indices of the cells, respectively. This setup assumes the current sensor configuration is $\left(x_{1},y_{1}\right),\left(x_{2},y_{2}\right),\cdots ,\left(x_{s},y_{s}\right)$.

#### 3.3.2. The GDOP Values Were Calculated

ToA positioning principle, combined with the trilateration method, is employed to determine the coordinates of each cell’s center point and to calculate the distance from each sensor to the center point ${\hat{p}}_{r}^{s}$:

(11) $$\left[\begin{array}{c} {\hat{p}}_{r}^{1}\\ {\hat{p}}_{r}^{2}\\ \vdots \\ {\hat{p}}_{r}^{n} \end{array}\right]=\left[\begin{array}{cccc} \frac{\partial \rho_{r}^{1}}{\partial e_{r}} & \frac{\partial \rho_{r}^{1}}{\partial n_{r}} & \frac{\partial \rho_{r}^{1}}{\partial u_{r}} & 1\\ \frac{\partial \rho_{r}^{2}}{\partial e_{r}} & \frac{\partial \rho_{r}^{2}}{\partial n_{r}} & \frac{\partial \rho_{r}^{2}}{\partial u_{r}} & 1\\ \vdots & \vdots & \vdots & \vdots \\ \frac{\partial \rho_{r}^{n}}{\partial e_{r}} & \frac{\partial \rho_{r}^{n}}{\partial n_{r}} & \frac{\partial \rho_{r}^{n}}{\partial u_{r}} & 1 \end{array}\right]\cdot \left[\begin{array}{c} {\hat{e}}_{r}\\ {\hat{n}}_{r}\\ {\hat{u}}_{r}\\ {\hat{d}}_{r} \end{array}\right]+\left[\begin{array}{c} -dt_{1}+l_{r}^{1}+T_{r}^{1}\\ -dt_{2}+l_{r}^{2}+T_{r}^{2}\\ \vdots \\ -dt_{n}+l_{r}^{n}+T_{r}^{n} \end{array}\right]$$

Here, $e_{r},n_{r},u_{r}$ represents the three-dimensional position of the measured object. ${dt}_{r}$ and ${dt}_{s}$ represent the clock error of the receiver clock and the satellite clock, respectively. $l_{r}^{s}$ and $T_{r}^{s}$ represents the ionospheric delay and tropospheric delay experienced by the navigation signal during propagation, respectively. ${\hat{p}}_{r}^{s}$ and $\left[\begin{array}{c} {\hat{e}}_{r},{\hat{n}}_{r},{\hat{u}}_{r},{\hat{dt}}_{r} \end{array}\right]$ represent the unbiased estimates of each pseudo-observed parameter and each unknown parameter, respectively.

The unbiased estimates of the observed parameters and the unknown parameters are denoted by vectors $\hat{p}$ and $\hat{x}$, respectively. The constant term in the system is represented by *l*. The system can be expressed as follows:

(12) $$\hat{p}=H^{T}\hat{x}+l$$

Expand ${\left(H^{T}\times H\right)}^{-1}$ for

(13) $${\left(H^{T}H\right)}^{-1}=\left[\begin{array}{cccc} D_{11} & D_{12} & D_{13} & D_{14}\\ D_{21} & D_{22} & D_{23} & D_{24}\\ D_{31} & D_{32} & D_{33} & D_{34}\\ D_{41} & D_{42} & D_{43} & D_{44} \end{array}\right]$$

GDOP values can alternatively be calculated using the formula

(14) $$GDOP=\sqrt{D_{11}+D_{22}+D_{33}+D_{44}}=\sqrt{\mathrm{tr}\left({\left(H^{T}H\right)}^{-1}\right)}$$

The GDOP is calculated for each cell $P_{ij}$, with the GDOP value for the entire region denoted as $G_{ij}$. This value is obtained by summing the GDOP values of all individual cells within the region, as represented by:

(15) $$G\left(\left(x_{1},y_{1}\right),\left(x_{2},y_{2}\right),\cdots ,\left(x_{s},y_{s}\right)\right)=\sum_{i}\sum_{j}G_{ij}$$

To assess the overall layout using GDOP values, the layout is considered optimized when the sum of GDOP values is minimized. The global minimum represents the optimal point.

#### 3.3.3. Layout Structure Optimization

- Define Initial Positions: Set the initial positions of the ultrasonic beacons and reference points within a 2 m × 2 m × 2 m cubic localization area. Position the five ultrasonic beacons at the initial 2D coordinates (0,0), (0,2), (1,1), (2,0), and (2,2). Randomly generate 100 reference points on the ground within this area.
- Initialize Parameters: Set the initial minimum GDOP sum to infinity and initialize the iteration counter to 10,000.
- Perform Iterations: Iterate until the number of iterations exceeds 10,000. In each iteration, randomly select a beacon and adjust its position by a step size of 0.01. After making these adjustments, calculate the GDOP for each reference point. Given the limited signal coverage of the ultrasonic beacons, select the three closest beacons to each reference point. If any of these beacons is more than 3 m from the reference point, assign a GDOP value of 10 to that reference point.
- Update Minimum GDOP: Calculate the sum of the GDOP values for all reference points. If this sum is less than the current minimum GDOP sum, update the minimum GDOP sum and the coordinates of the beacons accordingly.

## 4. Experiments and Results Analysis

To validate the effectiveness of the proposed ultrasonic array-based multi-source fusion indoor positioning system, a series of experiments were designed to assess the system’s positioning accuracy and robustness in complex environments. The experiments were conducted in a simulated underground mine environment, characterized by challenges such as multipath attenuation and signal obstruction. The experiments were divided into two parts: first, we compared different configurations of ultrasonic transmitter layouts to optimize the system’s Geometric Dilution of Precision (GDOP); second, we tested the system’s positioning performance under various obstruction conditions.

The experimental results demonstrate that with the optimal transmitter layout, the proposed system can achieve high-precision positioning with an error margin within 10 cm, even in the absence of satellite signals. Additionally, the system maintained robust performance under partial signal obstruction, though positioning accuracy declined under complete obstruction. These experiments confirm the system’s practicality and reliability in complex environments and provide a foundation for future optimizations.

**Figure 10:**
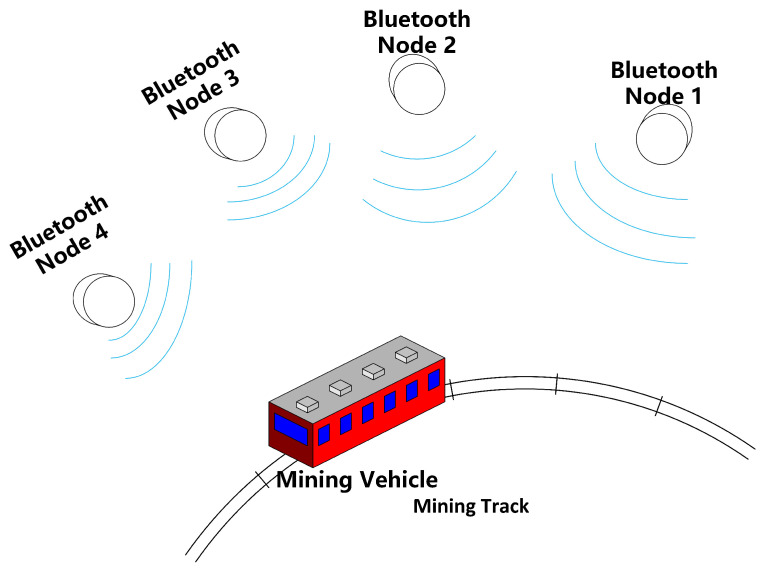
Bluetooth beacon operation inside the mine.

**Step 1:** Begin with a coarse localization of the Bluetooth beacon. As illustrated in Figure 10, the operation of the mine car on the mine shaft track is roughly positioned using Bluetooth.This step involves roughly estimating the position of the beacon to narrow down its location before proceeding with more precise measurements.

Establish a Bluetooth beacon network: Initially, deploy multiple Bluetooth beacons throughout the indoor environment. These beacons will emit signals that will be utilized in the subsequent positioning process to determine their locations accurately.

Establish the fingerprint database during the offline stage: The site is divided into a grid, and data points are collected at regular intervals. The position coordinates are correlated with the received signal strength indicator (RSSI) measurements to build a comprehensive fingerprint database. This database allows for the mapping of coordinates based on the RSSI fingerprints.

(16) $$\left(RSSI_{i},RSSI_{2},RSSI_{3},\cdots ,RSSI_{n}\right)↔\left(x,y,z\right)$$

Online positioning stage: The optimal location information is determined by analyzing the signal strength data in conjunction with the fingerprint database. This analysis helps to accurately map and identify the position based on the collected RSSI measurements.

**Step 2:** Perform ultrasonic precision calibration to ensure accurate measurements and reliable positioning. This step involves adjusting and fine-tuning the ultrasonic system to correct for any inaccuracies and improve overall precision.

The four ultrasonic emission terminals were mounted on a tripod at a height of 1.5 m in a square layout with dimensions 2.4 m by 2.4 m. The ultrasonic transmitters are controlled in a clockwise sequence. When transmitter 1 emits an RF signal, both the receiver and transmitter 2 receive the signal simultaneously, allowing the receiver to determine the distance and angle relative to transmitter 1. Subsequently, transmitter 2 switches from receiving to transmitting mode and sends a signal to transmitter 3, enabling a clockwise self-circulation sequence of 1-2-3-4-1. The absolute position is then calculated by analyzing the distance and angle information obtained from all four emitters.

**Figure 11:**
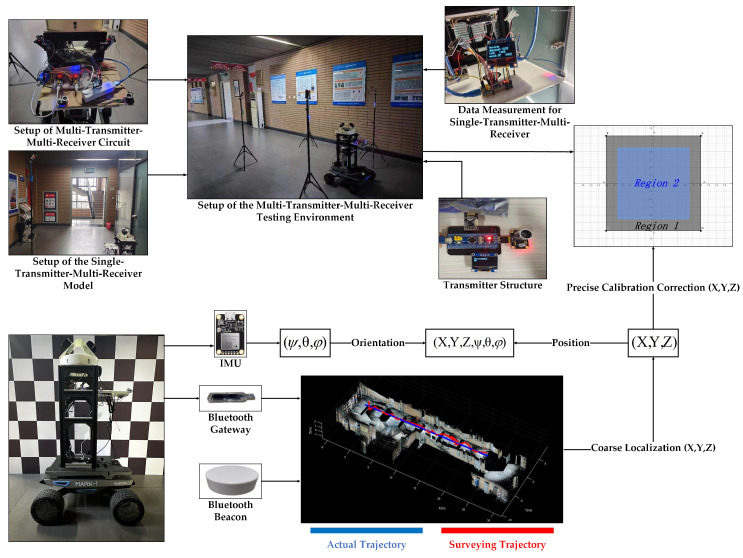
Setup of the ultrasonic testing environment.

As shown in Figure 11, a Bluetooth gateway and an inertial navigation module are mounted on the mobile cart. Bluetooth beacons are deployed every 5 m in the corridor for coarse positioning, with an ultrasonic precision calibration network set up around each beacon. An ultrasonic receiving array is mounted around the cart. During the experiment, a network of four ultrasonic transmitters was established, with the transmitters positioned at vertices *A*, *B*, *C*, and *D*, forming a square layout with dimensions 2.4 m by 2.4 m, referred to as Region One. The coordinate system was established with the center of this square as the origin. The following data present the measurement results within this coordinate system (unit: mm).

### 4.1. Comparison of the Single-Transmitter-Multi-Receiver Model with the Traditional Single-Transmitter-Single-Receiver Model

In the experimental environment, while the vehicle is in motion, a multi-receiver model is employed to accurately calculate the distance and angle between the vehicle and a single transmitter, thereby deducing their relative positions. The simulation involves the vehicle moving past the ranging transmitter nodes to assess the ranging accuracy under different conditions, including no occlusion, partial occlusion, and complete occlusion.

**Figure 12:**
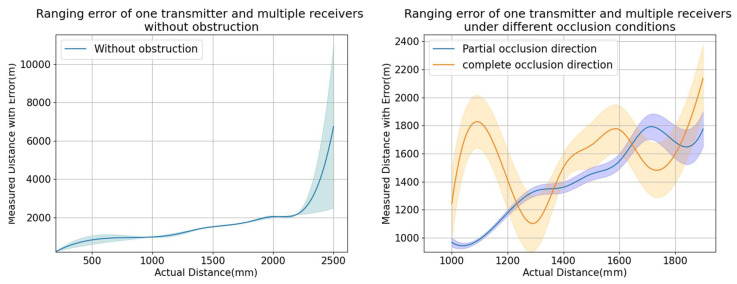
Range measurement results for the single-transmitter-multi-receiver model.

As illustrated in Figure 12, the overall ranging performance of the multi-receiver model is controlled to within 50 mm to ensure measurement accuracy. In the no-occlusion scenario shown on the left side of the figure, the optimal ranging distance is within 2 m, where accuracy is at its best. Beyond 2 m, the accuracy significantly deteriorates. The multi-receiver model maintains an overall ranging distance within a 2-m radius, with an error margin of ±25 mm in unobstructed conditions. On the right side of the figure, under partial occlusion, the ranging error is within ±50 mm. In cases of complete occlusion, the positioning system becomes ineffective, and the range errors exhibit unpredictability and lack of regularity.

Compared to the traditional single-transmitter-single-receiver model, which typically maintains ranging accuracy within 10 mm to 50 mm under no-occlusion conditions, this model experiences significant offset and error issues under partial occlusion. In contrast, the multi-receiver model’s accuracy decreases by approximately 5% to 10% under no occlusion, but it remains relatively stable in various occlusion scenarios. Although this approach sacrifices some range accuracy, it enhances the system’s anti-interference capabilities and overall robustness in complex environments, effectively addressing the limitations of the traditional model under partial occlusion. However, neither model performs effectively under complete occlusion. To overcome these limitations, future work will focus on developing a multi-transmitter-multi-receiver system to address the challenges of ranging under complete occlusion conditions.

In future work, we plan to improve ranging under occlusion conditions by leveraging techniques such as analyzing reflected signals via multipath effects to estimate the target’s location, deploying relay devices or reflectors to help signals bypass obstacles, fusing data from multiple sensors (like sound waves, radio waves, and infrared) to enhance the system’s robustness, and using environmental models combined with historical data to predict the position of occluded objects, thus reducing reliance on direct ranging. This approach aims to address challenges in environments like mines, where ultrasonic waves may still have limited penetration through opaque walls.

### 4.2. Comparison of the Multi-Transmitter-Multi-Receiver Model with Traditional Models

During signal processing, time-division signal recognition technology is employed to differentiate and identify signals from various transmitters. Using the four distance measurements obtained, a trilateration algorithm is applied to determine the absolute positions of the emitters and generate the final positioning results.

**Figure 13:**
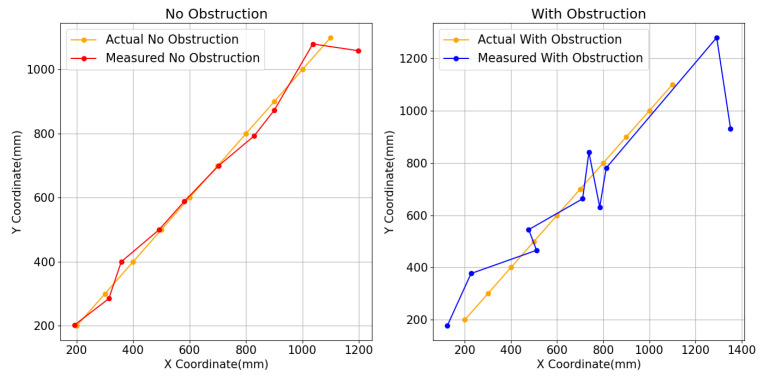
Range measurement error analysis for the multi-transmitter-multi-receiver model.

As shown in Figure 11, after thorough verification of the experimental range, it was determined that within Region 2—a square area measuring 1.8 m by 1.8 m, centered on Region 1—the range performance was optimal due to the specific angles of the emitters. The measurement results from Region 2 have been compiled into an experimental data table, and a detailed data analysis is provided in Figure 13 below.

Under no-occlusion conditions, the equipment’s ranging accuracy can achieve a precision within 50 mm. However, as the device moves closer to the transmitter, ranging accuracy tends to decrease. This decrease is closely related to the angular factors of the transmitter. Specifically, when the device is near a particular transmitter, ranging errors in the other three directions tend to increase significantly, thereby reducing overall accuracy. In cases where one direction is completely blocked, the device can still achieve better positioning accuracy by relying on ranging information from the remaining three directions. Under such conditions, the device’s ranging accuracy is typically maintained within 150 mm.

In the multi-transmitter-multi-receiver process, the ranging process between each transmitter and the vehicle involves using the cosine theorem twice within the multi-receiver model to calculate the angle between the vehicle’s centerline and each transmitter. These angles, combined with data from the inertial navigation system, are used to derive and correct the vehicle’s attitude parameters, including orientation, pitch, and roll. Additionally, the inertial navigation module is employed to further refine and plan the vehicle’s trajectory by correcting its operational state.

#### 4.2.1. Comparison with the Single-Transmitter-Multi-Receiver System

Compared to the single-transmitter-multi-receiver system, the multi-transmitter-multi-receiver system exhibits superior ranging performance and enhanced anti-interference capabilities, especially in cases of complete occlusion. When confronted with complex environmental conditions, such as when a particular direction is completely blocked, the system assigns a maximum value to that direction and discards it as an outlier. Positioning calculations are then performed using the remaining three smaller values. This method allows the system to achieve relatively ideal positioning results despite the occlusion. However, the increased internal calculation complexity of the multi-transmitter-multi-receiver system may lead to processing errors, affecting accuracy in the absence of interference and potentially increasing ranging errors. Overall, this approach effectively addresses the issue of ranging difficulties in complex environments with occlusions, maintaining good positioning accuracy.

#### 4.2.2. Comparison with the Multi-Transmitter-Single-Receiver System

**Figure 14:**
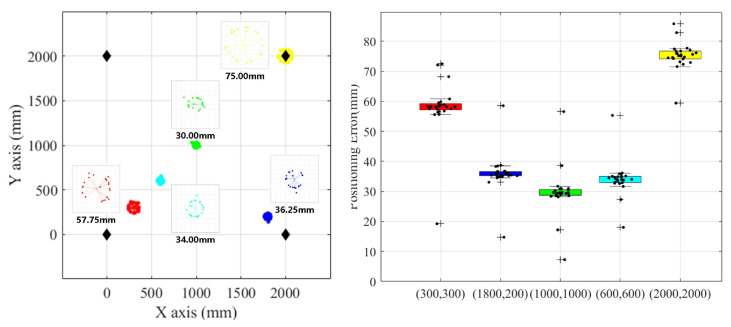
Range measurement error for the multi-transmitter-single-receiver system.

In the same transmitter environment, a single receiver was mounted on a vehicle, and measurements were taken at various nodes to compare the results with those of the multi-transmitter-multi-receiver system. The measurements were also verified under occlusion conditions. The experimental results are presented in Figure 14. In Figure 14, different colors in the left image represent different ranging positions, and black diamonds represent transmitter positions. The black dots in the image on the right represent distance measurement errors, while different color bands represent the measurement error ranges at their respective measurement positions.

According to the ranging data, the measurement error increases progressively near the edge of the coverage area. In an unobstructed environment, the ranging accuracy remains within 100 mm. However, in the presence of occlusion, the positioning function may fail completely due to the lack of ranging data in a single direction, leading to significant bias in the overall positioning results. In contrast, the multi-transmitter-multi-receiver system demonstrates strong resistance to multipath interference in occluded environments. It can provide relatively accurate positioning results even under complete occlusion, maintaining positioning accuracy within an acceptable range.

### 4.3. Comparison of Positioning Performance before and after GDOP Improvement

Traditional layout methods are generally classified into several types: closed polygons (such as rectangles and parallelograms), circles, and spiral shapes. Among these, closed polygon layouts are often more practical for indoor environments compared to circular and spiral layouts. Experimental verification of various common polygon layouts has been conducted to assess their effectiveness.

#### 4.3.1. Theoretical Verification

When comparing the number of base stations, a range of three to seven base stations was evaluated. Using the orthogonal experimental method, with all base stations positioned at the same height, the relevant coordinate data were input into the MATLAB system for GDOP calculations. The results revealed that a layout with three base stations resulted in dense singularities in the target area, indicating that a three-base station configuration is insufficient for high-precision 3D positioning.

Increasing the number of base stations to four significantly improved the GDOP values, though the improvement trend gradually flattened with additional base stations. After considering the balance between cost-effectiveness and positioning accuracy, the four-base station layout was chosen as the optimal solution.

To further optimize the geometric layout of the base stations, we designed five different layout schemes: rectangular, parallelogram, diamond, trapezoidal, and triangular. The coordinate data for each layout scheme were input into the MATLAB system for GDOP calculations. Table 1 presents the GDOP values for each layout scheme.

Analysis reveals that compared to the traditional rectangular layout, a parallelogram layout with four base stations achieves the lowest average GDOP value. Additionally, its maximum and minimum GDOP values are among the best across all five layout schemes. This indicates that the parallelogram layout offers significant advantages over the conventional rectangular layout. Further observation shows that the benefit of the parallelogram layout is more pronounced when it includes smaller internal angles. Based on this analysis, we proceed to validate the parallelogram layout as the preferred geometric scheme for base stations over the traditional rectangular layout.

#### 4.3.2. Experimental Demonstration

In an experimental environment within a 2 m × 2 m × 2 m frame placed in a room, the ultrasonic transmitting device was mounted at the top of the frame, 2 m above the ground, while the ultrasonic receiving device was placed on the floor. The positioning system was tested under four conditions:

**Layout1 GDOP:** The ultrasonic transmitters were positioned at (0,0), (50,100), (100,0), and (150,100) in a GDOP-optimized arrangement.

**Layout2 Non-GDOP:** The ultrasonic transmitters were positioned at (0,0), (200,0), (0,200), and (200,200) without GDOP optimization.

**Layout3 GDOP with Wall-Mounted Beacons:** Two of the ultrasonic beacons were attached to the walls, following the GDOP layout principles.

**Layout4 Non-GDOP with Wall-Mounted Beacons:** Two of the ultrasonic beacons were attached to the walls without GDOP optimization.

**Figure 15:**
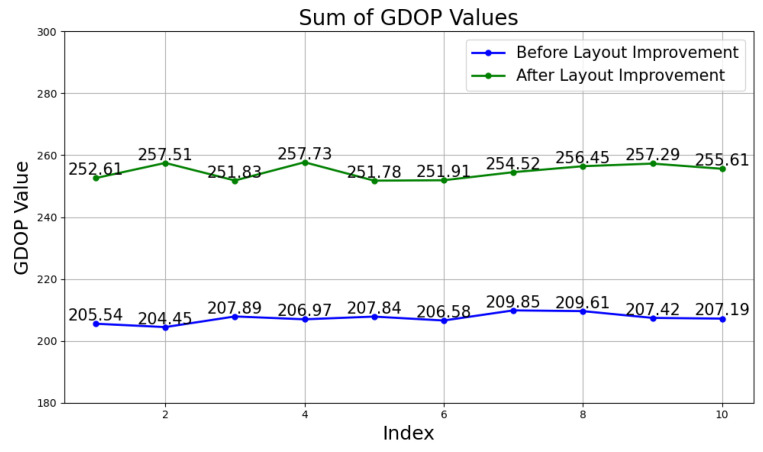
Sum of GDOP values for reference points before and after layout improvement.

The GDOP values for layouts (1) and (2) were simulated using the MATLAB platform. The results are illustrated in Figure 15.

**Figure 16:**
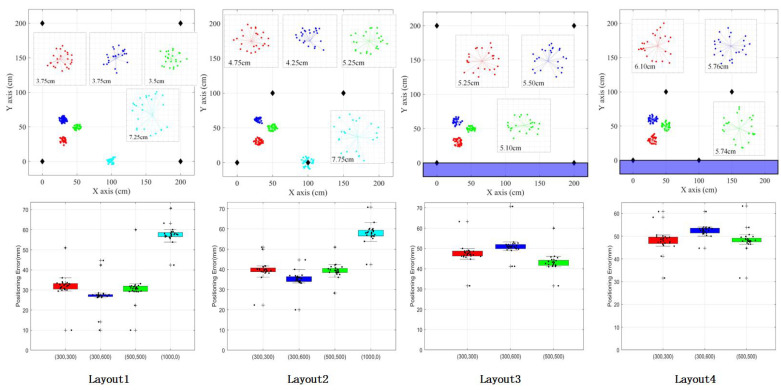
Overall GDOP calculation results.

As depicted in Figure 16, the experimental setup is configured as follows:

Control Subsystem: Positioned at any location on the ground within a 2 m × 2 m frame. Ultrasonic Transmitter Subsystem: Mounted at the top of the frame. Ultrasonic Receiving Subsystem: Located on the ground within the frame. The control subsystem communicates with the ultrasonic transmitter subsystem using a radio frequency module. Meanwhile, ultrasonic receivers 1 and 2 send data to the receiving host via a serial port.

To ensure adequate coverage, four ultrasonic beacons were placed within the 2 m × 2 m area at predefined coordinates. For experimental accuracy, four distinct points within the environment were selected, and measurements were conducted four times for each layout configuration.

Initially, the coordinate solutions for a stationary target were verified to assess system performance. The resulting positioning data are illustrated in Figure 16.

In Figure 16, the different colors in the upper image represent different ranging positions, the black diamond represents the transmitter position, and the blue area represents the wall obstructing the transmission of ultrasonic waves. The black dots in the image below represent distance measurement errors, and different color bands represent the measurement error ranges at their respective measurement positions.

**Results and Analysis:** The positioning system demonstrates that the positioning error is generally maintained within 8 cm, with a maximum observed error of 10 cm at the edge of the 2 m × 2 m frame. Based on the calculations shown in Figure 16 for the GDOP-based layout, the average positioning error is 4.7, with a variance of 6.94 and a standard deviation of 2.41.

**GDOP Layout:** Compared to layouts without GDOP optimization, the GDOP layout achieves higher positioning accuracy, meeting the high precision indoor positioning requirements outlined in this study. When beacons are mounted on the walls, a ranging blind spot emerges at the edge of the 2 m × 2 m area, leading to unsolvable coordinates and larger positioning errors.

**Layout Performance:** Excluding the special case of edge limitations, the average error for Layout (1) is the smallest, aligning with the improved experimental requirements. The optimized parallelogram layout, in particular, offers a notable reduction in average error compared to the traditional rectangular layout, with an average error decrease of 1 cm. This layout also shows an approximate 20% improvement in performance and performs well in both wall-mounted and free-space environments.

**Figure 17:**
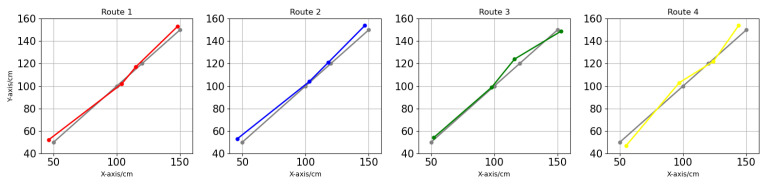
Localization route map.

**Next Steps:** To further validate system performance, a dynamic positioning experiment will be conducted using Layout (1) (GDOP improvement, free space). The experiment will involve recording real-time position data of the receiving end during irregular movements and comparing this data with the actual position to evaluate the system’s navigation accuracy. The positioning roadmap for this experiment is illustrated in Figure 17. Different colors represent different paths of multiple measurements.

Based on the dynamic localization error data, the average error is calculated to be 5.00 cm, with a maximum localization error of 10 cm. The standard deviation is 3.2, and the variance is 11. A comparison with static positioning error data is shown in Table 2 below.

Dynamic positioning experiments reveal increased errors compared to static positioning, with an average error of 5.00 cm, a maximum error of 10 cm, and a standard deviation of 3.2 cm. This discrepancy arises because the vehicle’s movement during dynamic positioning introduces additional errors. The system’s measurement and processing delays exacerbate this issue, as continuous movement leads to positional shifts that are not immediately captured by the system. Consequently, the combination of these delays and movement causes ranging errors that increase average localization error and variability, unlike static positioning where the constant position minimizes such errors.

### 4.4. Summary of This Chapter

In our study, the combination of Bluetooth and ultrasonic showed good performance, particularly in environments without obstructions. However, to further enhance the robustness of the system, future work could consider incorporating IMU and PDR modules to improve dynamic tracking capabilities, or introducing UWB technology to enhance positioning accuracy, especially in complex environments such as mines.

In the experimental results, we observed significant variations in positioning accuracy under different conditions. The proposed method maintained high positioning accuracy in complex environments, particularly under partial signal obstruction, where the system continued to perform stably. This improvement in performance can be attributed to several key strategies:

Firstly, the introduction of the Geometric Dilution of Precision (GDOP) optimization algorithm played a crucial role. By optimizing the layout of the transmitters, the system’s geometric errors were reduced, enabling consistent positioning accuracy across different locations and angles. The experiments demonstrated that after GDOP optimization, the system’s positioning accuracy significantly improved, with error margins reduced to within 10 cm.

Secondly, the method employed a multi-source data fusion strategy, integrating Bluetooth, ultrasonic, and inertial navigation data. This fusion not only enhanced the reliability of the positioning data but also mitigated the negative impacts of signal reflection and attenuation by compensating for multipath effects. This approach was particularly effective in complex environments such as mines, where multi-source fusion helped alleviate accuracy degradation caused by the failure or instability of individual signal sources.

Lastly, the proposed multi-transmitter-multi-receiver processing algorithm enhanced the system’s robustness in complex environments. By filtering out low-confidence measurements, this strategy reduced positioning errors caused by noise and interference, which was especially critical in scenarios with partial signal obstruction and significant reflections.

In summary, these strategies collectively contributed to a significant enhancement in the system’s positioning accuracy and robustness across various complex environments, thereby validating the effectiveness and practicality of the proposed method.

## 5. Conclusions

The proposed ultrasonic array-based multi-source fusion indoor positioning system has demonstrated superior performance in complex environments such as underground mines, where traditional localization techniques often struggle. This system effectively addresses challenges posed by multipath attenuation and signal reliability, achieving high-precision positioning even in the absence of satellite signals. However, the effectiveness of the system is contingent upon the careful placement of Bluetooth beacons and ultrasonic transmitters, as environmental obstacles can significantly impact signal propagation and accuracy, especially under conditions of complete occlusion.

Looking ahead, future research will focus on refining the layout algorithms to better manage signal attenuation and occlusion effects, thereby extending the system’s applicability to larger and more complex environments. Additionally, by integrating complementary sensing technologies such as 5G communication, Internet of Things (IoT) devices, and LiDAR, the system’s positioning accuracy and real-time responsiveness can be further enhanced. We also envision expanding the application of this technology to dynamic environments, including mobile robot navigation, autonomous vehicle positioning, and dynamic asset tracking.

With continued advancements in sensor integration and algorithm optimization, this system holds significant potential for deployment across a wide range of industrial and commercial fields, providing a robust foundation for more intelligent and automated environmental management.

## Figures and Tables

**Table 1:** GDOP calculation for various schemes.

Different Scenarios	Minimum	Maximum	Mean
Rectangle	0.30612	27477	26.8
Parallelogram	0.6762	9.0282	2.2632
Lozenge	0.0946	8.3466	2.4585
Trapezium	0.305	72.3004	8.5788
Triangle	0.2779	9.1308	2.5508

**Table 2:** Comparison of dynamic and static positioning.

	Positioning Error: Mean (cm)	Variance	Standard Deviation
Static Positioning	4.7	6.94	2.41
Dynamic Positioning	5	11	3.2

## Data Availability

The original contributions presented in the study are included in the article, further inquiries can be directed to the corresponding author/s.
